# *CgSTE11* mediates cross tolerance to multiple environmental stressors in *Candida glabrata*

**DOI:** 10.1038/s41598-019-53593-5

**Published:** 2019-11-19

**Authors:** Mian Huang, Jibran Khan, Manpreet Kaur, Julian Daniel Torres Vanega, Orlando Andres Aguilar Patiño, Anand K. Ramasubramanian, Katy C. Kao

**Affiliations:** 1 Artie McFerrin Department of Chemical Engineering, Texas A&M University, College Station, Texas 77843 United States of America; 2 Department of Biology, Texas A&M University, College Station, Texas 77843 United States of America; 3 Department of Chemical and Materials Engineering, San José State University, San José, CA 95192 United States of America; 4 Department of Chemical Engineering, Industrial University of Santander, Bucaramanga, Colombia

**Keywords:** Genetics, Fungi

## Abstract

*Candida glabrata* is a human commensal and an opportunistic human fungal pathogen. It is more closely related to the model yeast *Saccharomyces cerevisiae* than other *Candida spp*. Compared with *S*. *cerevisiae*, *C*. *glabrata* exhibits higher innate tolerance to various environmental stressors, including hyperthermal stress. Here we investigate the molecular mechanisms of *C*. *glabrata* adaptation to heat stress via adaptive laboratory evolution. We show that all parallel evolved populations readily adapt to hyperthermal challenge (from 47 °C to 50 °C) and exhibit convergence in evolved phenotypes with extensive cross-tolerance to various other environmental stressors such as oxidants, acids, and alcohols. Genome resequencing identified fixation of mutations in *CgSTE11* in all parallel evolved populations. The *CgSTE11* homolog in *S*. *cerevisiae* plays crucial roles in various mitogen-activated protein kinase (MAPK) signaling pathways, but its role is less understood in *C*. *glabrata*. Subsequent verification confirmed that *CgSTE11* is important in hyperthermal tolerance and the observed extensive cross-tolerance to other environmental stressors. These results support the hypothesis that *CgSTE11* mediates cross-talks between MAPK signaling pathways in *C*. *glabrata* in response to environmental challenges.

## Introduction

The ability to adapt to changing environments is essential for microbial survival. Temperature is an environmental variable that has profound impacts on the physiology of living organisms. If the temperature falls outside of the range an organism could tolerate, depending on the duration of exposure, damage to cellular components will ensue, eventually leading to cell death^1,2^. In yeast, response to heat stress is most well studied in *S*. *cerevisiae*, in which the mitogen-activated protein kinase (MAPK) signaling pathways play critical roles^3^. There are currently 5 known MAPK signaling pathways in *S*. *cerevisiae*. In response to elevated temperatures, the cell wall integrity (CWI) and high-osmolarity glycerol (HOG) pathways^4–6^ are activated. The HOG pathway involves two independent branches, the Sho1 branch that involves osmosensors Hrk1 and Msb2^7^ and Sho1^8^, and the Sln1 branch with Sln1 as the osmosensor^9^. Heat stress activates the CWI pathway by two transmembrane sensors, Slg1^10^ and Mid2^11^; deletion of components in this signaling cascade have been shown to result in slow growth at 30 °C and cell lysis at 37 °C^5,12^. In addition, heat stress also activates Hog1, the MAPK of HOG pathway, via the Sho1 branch, but not the Sln1 branch^6^. The heat stress-activated Hog1 contributes to the rapid cell recovery from the challenge; interestingly, it is not localized in the nucleus after activation in response to heat, suggesting that the phenotypic effects are not due to its direct downstream transcriptional regulations^6^. The cross-talk between the HOG and CWI signaling pathways upon heat stress was recently elucidated to be mediated by Nst1 in *S*. *cerevisiae*^13^. In addition to signaling pathways, the heat stress response (HSR) in *S*. *cerevisiae* involves several transcription factors, including Hsf1, Msn2, and Msn4, and their downstream targets^14–16^. Temperature sensing mechanisms have also been proposed, such as the membrane fluidity change, unfolded protein responses, and RNA thermometer (reviewed in^17^).

*C*. *glabrata* is an opportunistic human fungal pathogen and is more closely related to *S*. *cerevisiae* than other *Candida spp*. It exhibits a higher inherent tolerance to various environmental stressors such as heat, organic acids, low pH, and oxidant, compared with *S*. *cerevisiae*^18,19^. Homologs of many central components of the MAPK pathways (*e*.*g*. HOG and CWI pathway) and major regulators of general stress response (*e*.*g*. Msn2, and Msn4) in *S*. *cerevisiae* are found in *C*. *glabrata* based on their amino acid identities or through the few existing functional characterizations^18,20^. Prior study found growth to be unaffected by deletion of *SHO1*, the gene encoding the osmosensor and scaffolding protein within the Sho1 branch of HOG pathway^8^, at 37 °C, but is significantly inhibited at 42 °C, demonstrating that the Sho1 branch of HOG signaling in *C*. *glabrata* influences cellular tolerance to high temperature^21^. The *C*. *glabrata* Msn2 and Msn4 orthologues have also been found to be involved in the regulation of gene expression in response to multiple stresses, including heat, osmotic, oxidative, and glucose starvation^20^. While the few existing studies have identified several cellular components involved in stress tolerance in *C*. *glabrata* based on knowledge in *S*. *cerevisiae*, the regulatory and signaling pathways involved in response to environmental stressors, including heat, are still largely unknown in this important yeast.

In this work, we harnessed the power of adaptive laboratory evolution to uncover the molecular mechanisms of *C*. *glabrata* adaptation to heat stress using periodic hyperthermal challenge. The results showed that *C*. *glabrata* readily adapts to step-wise increases in heat challenge (from 47 °C to 50 °C). Interestingly, the hyperthermal tolerant mutants isolated from parallel populations exhibited convergence in evolved phenotypes with extensive cross-tolerance to other environmental stressors. Mutations in the MAPK kinase kinase *CgSTE11* were found in all parallel populations, suggesting the importance of this gene in hyperthermal tolerance. Subsequent verification via site-directed mutagenesis confirmed that mutations in *CgSTE11* played a major role in the hyperthermal tolerance and the observed cross-tolerance to other environmental stressors, providing strong evidence that *CgSTE11* mediates cross-talks between MAPK signaling pathways in *C*. *glabrata* in response to environmental stressors.

## Results

### Adaptive evolution of *C*. *glabrata* in periodic hyperthermal challenge

**Figure 1:**
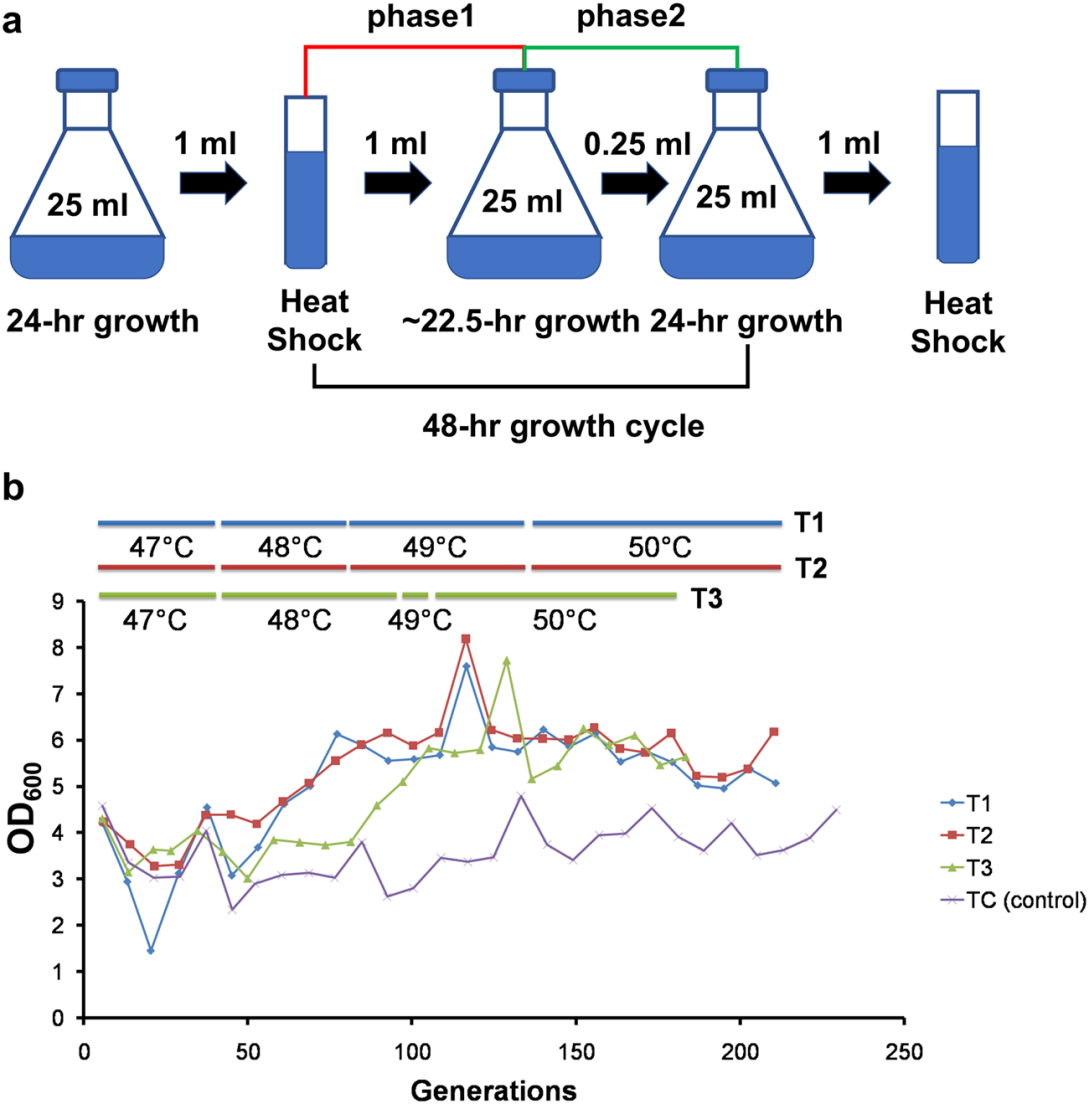
Adaptive laboratory evolution of *C*. *glabrata* to hyperthermal challenge. (**a**) The two-phase periodic heat challenge strategy used for adaptive evolution of *C*. *glabrata*. (**b**) Changes in the population cell density (OD_600_) during the adaptive evolution of *C*. *glabrata*. The temperature used for periodic hyperthermal challenge are shown as colored bars (blue for T1, red for T2, and green for T3) above the graph. The control population TC was transferred in the absence of periodic hyperthermal challenge.

Three parallel populations (T1, T2, and T3) were evolved in YNB medium at elevated temperatures via serial batch transfers for more than 180 generations. Visualizing evolution in real-time (VERT)^22^ was used to track the population dynamics during evolution using two otherwise isogenic strains that are labeled with either yellow fluorescent protein (YFP) or green fluorescent protein (GFP). An adaptive evolutionary strategy using periodic challenge at high temperatures (≥47 °C) was employed (see Fig. 1a and Materials and Methods for details). A control population (TC) serially transferred at 30 °C without periodic hyperthermal challenge was included. Whenever a significant increase in cell growth (based on OD_600_ measurements at the end of Phase 1) was observed during the course of evolution (Fig. 1b), the viability of evolving population after heat exposure at various temperatures was measured and compared with the parental strains to determine whether the population exhibited improved hyperthermal tolerance (see Materials and Methods for details). If an improvement was observed, the selective pressure (temperature) was ramped up accordingly.

The evolutionary dynamics of all populations were estimated by quantifying the relative proportions of GFP- and YFP-colored subpopulations (Figs S1–S4). Any increase in the relative proportion of a colored-subpopulation is suggestive of the emergence and expansion of hyperthermal tolerant mutants within the population (an adaptive event). In populations T1 and T2, rapid sweeps by the GFP-colored subpopulation were observed early on during the evolution. Interestingly, there was a lack of significant increases in OD_600_ observed in T1 and T2 populations before generation ~37 even though expansion was observed in the GFP-subpopulations, suggesting that VERT is more sensitive in detecting adaptive events. However, due to the rapid sweep of one of the colored-subpopulations in 2 out of 3 parallel populations, use of VERT data to identify subsequent adaptive events in the population was not possible, thus increases in OD_600_ values were used instead. In population T3, the expansion and contractions of the GFP and YFP-colored subpopulations correlated fairly well with the OD_600_ data.

### Phenotypic characterizations of adaptive mutants

**Figure 2:**
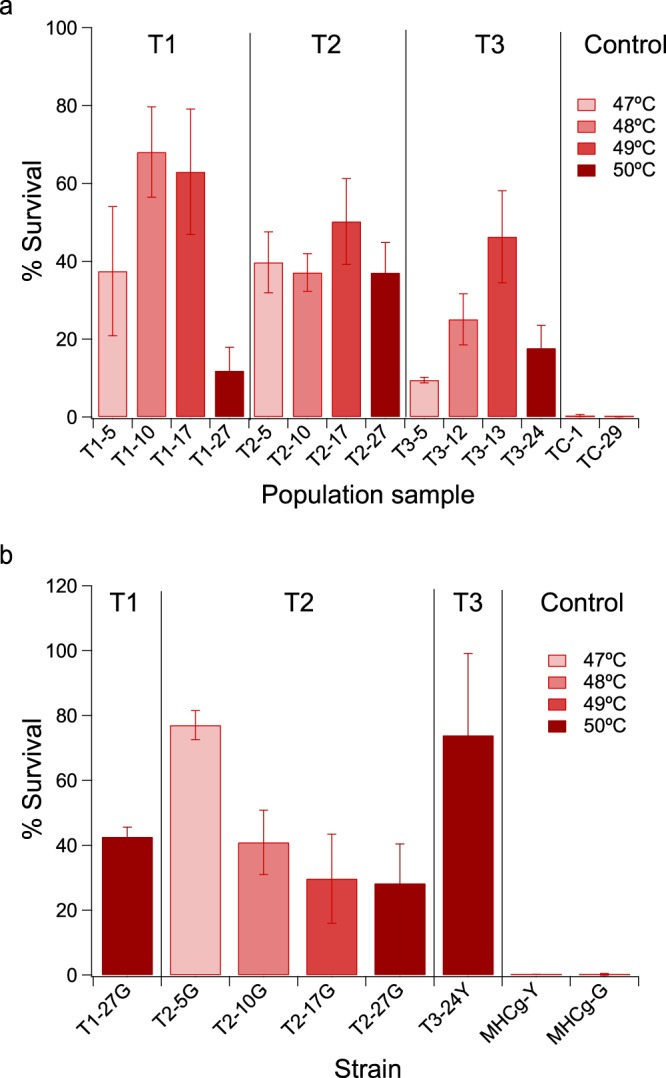
The survival rates of evolved populations (**a**) and isolated adaptive mutants (**b**) after 1-hr heat shock treatments at the specified temperatures. The data shown is the mean survival rates based on three biological replicates. The controls TC-1 (1^st^ sample from the control population), TC-29 (last sample from the control population), and parental strains MHCg-Y and MHCg-G were challenged at 47 °C.

At the end of the evolution experiment, the population samples prior to each ramp-up in selective pressure were revived from frozen stocks. The viability of each population after one-hour exposure to the temperature that the time-course sample was challenged with during evolution was quantified. All populations tested gained significantly improved tolerance to heat challenge (Fig. 2a). Population T2–27 (from population T2, sample 27) exhibited the highest thermotolerance with an average viability of ~37% after heat shock at 50 °C for 1 hour. In contrast, the average viability of the parental strains, MHCg-Y and MHCg-G, were ~0.35% after challenged at a much lower temperature (47 °C) for 1 hour. Population T2 was chosen for more detailed time course characterizations.

**Figure 3:**
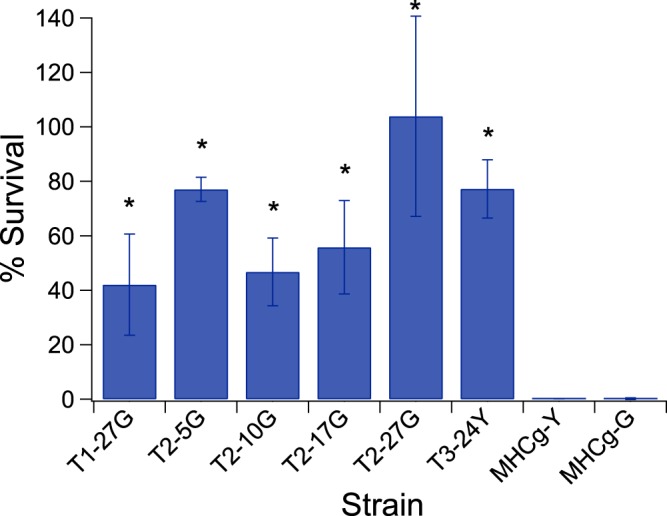
The survival rates of adaptive mutants and parental strains (MHCg-Y and MHCg-G) after 1-hr heat shock treatments at 47 °C. The data shown is the mean survival rates from at least three biological replicates. Asterisks indicate that the survival rates of adaptive mutants are statistically different from the parental strain, MHCg-G or MHCg-Y (p-value < 0.05, unpaired student t test with unequal variance).

Time course samples from population T2 and the end-point samples from T1 and T3 were selected for further phenotypic characterizations. Adaptive mutants T2-5G, T2-10G, T2-17G, and T2-27G were isolated from time points immediately prior to each ramp-up in selective pressure, from populations T2-5, T2-10, T2-17, and T2-27, respectively (Fig. S3). Adaptive mutants T1-27G and T3-24Y were isolated from the last time point samples of populations T1 and T3, respectively. Each adaptive mutant was analyzed for tolerance (survival) to 1-hour heat shock at the temperature at which they were challenged during the evolution. We observed significant increases in the viability after the heat challenge for all adaptive mutants tested when compared to the parental strains, MHCg-Y and MHCg-G (Fig. 2b). Among all the mutants tested, strain T3-24Y exhibited the highest viability after heat shock at 50 °C, with an average survival rate of ~74%. The controls (MHCg-Y and MHCg-G) in this experiment exhibited survival rates of ~0.2% after heat challenge at 47 °C for 1 hour. In addition, we also measured the viability of all the mutants after a 1 hour challenged at 47 °C; the data showed that all of the mutants exhibited much higher survival rates compared to the parental strains (Fig. 3).

**Figure 4:**
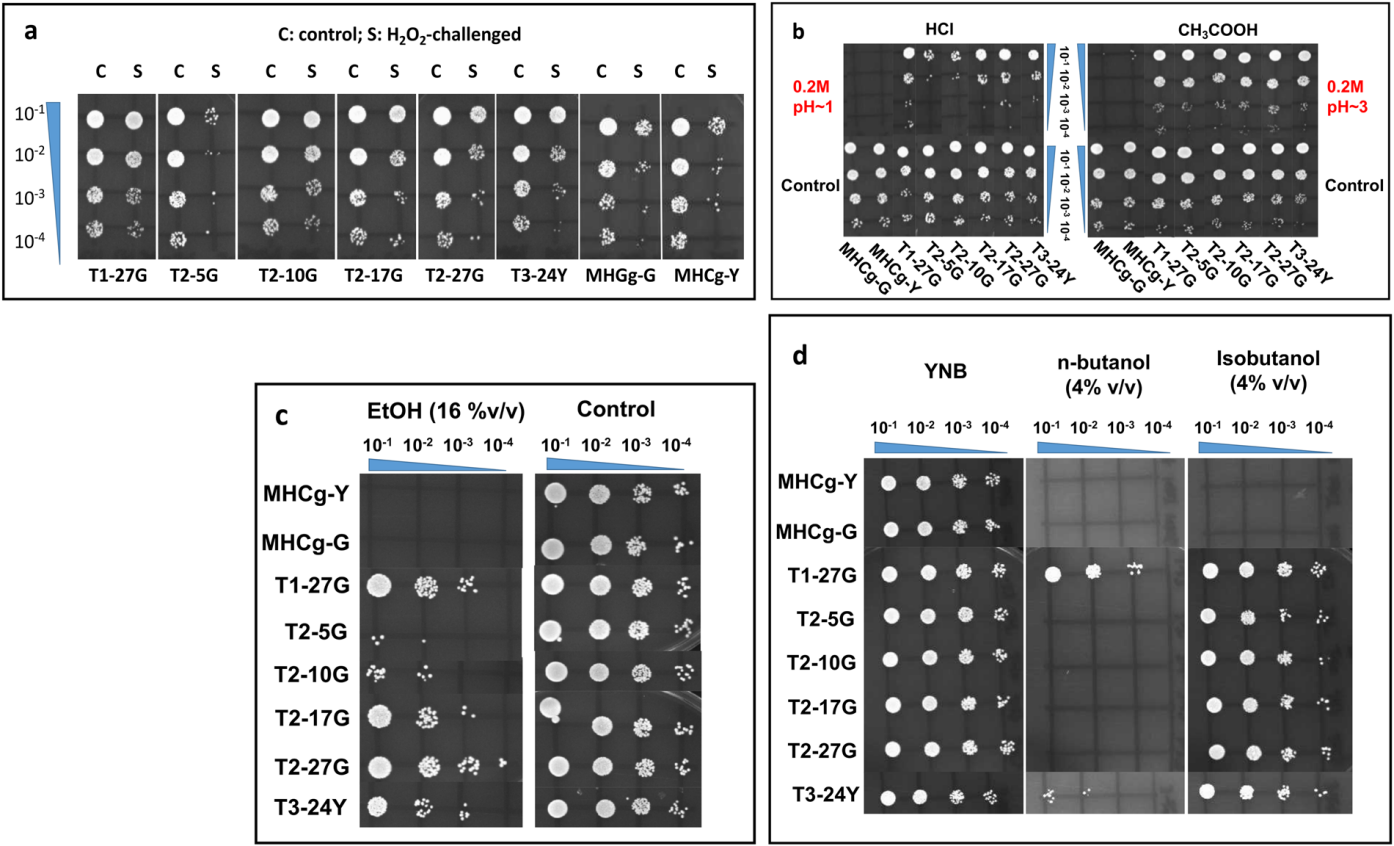
Cross-tolerance of isolated adaptive mutants and parental strains (MHCg-Y and MHCg-G) to H_2_O_2_ (**a**), HCl and acetic acid (**b**), ethanol (**c**), n-butanol and isobutanol (**d**). Each strain was subjected to 1-hour exposure to specified stressor, serial diluted, and plated on YNB medium supplemented with 2% dextrose at 30 °C for two days.

Cases of cross-tolerance to additional stressors were observed in prior adaptive laboratory evolution studies in other species (e.g. *Escherichia coli*, *Salmonella typhimurium*, and *Corynebacterium glutamicum*)^23–25^. For example, positive and negative cross-stress protection were observed in laboratory evolved *E*. *coli*^24^; and *Corynebacterium glutamicum* evolved under thermal stress also exhibited cross-tolerance to isobutanol^25^. Thus, we hypothesized that a genotype optimized for one phenotype (mutant evolved for tolerance to one stressor) may exhibit cross-benefit to other compatible phenotypes (cross-tolerance to other stressors). The evolved heat tolerant mutants were tested against a wide range of stressors, including oxidative stress (H_2_O_2_), acids (HCl and acetic acid), osmotic (glucose and sorbitol), antifungal drugs (amphotericin B and fluconazole), and solvents (ethanol, 1-butanol, iso-butanol), to assess their level of cross-tolerance to other stressors. Results are shown in Table 1 and Fig. 4. Compared to the parental strains, the evolved mutants exhibited significantly improved tolerance to many of these stressors. For some stressors tested (H_2_O_2_ and HCl), the results showed a gradual increase in the level of cross-tolerance to these stressors over time during the adaptive evolution to hyperthermal challenge in population T2. These results suggest that adaptation to thermal stress in *C*. *glabrata* may share similar mechanisms with adaptation to other stressors, possibly through common key components in stress sensing, signal transduction, and stress response.

**Table 1:** Summary of cross-tolerance of isolated mutants.

Strain ID	Oxidative Shock	Acid Shock		Solvent Shock			Osmotic Stress		Antibiotics	
	H_2_O_2_ (100 mM)	HCl (pH~1)	Acetic acid (pH~3)	EtOH (16%, v/v)	1-butanol (4%, v/v)	Isobutanol (4%, v/v)	Glucose (30%, w/v)	Sorbitol (2 M)	AmB (0.25~32 µg/mL)	Flu (1~128 µg/mL)
T1-27G	++	>++++	++++	>+++	>+++	>++++	N	N	N	N
T2-5G	N	>++	+++	>+	N	>++++	N	N	N	N
T2-10G	++	>++	+++	>++	N	>++++	N	N	N	N
T2-17G	++	>++	+++	>+++	N	>++++	N	N	N	N
T2-27G	+	>+++	++++	>++++	N	>++++	N	N	N	N
T3-24Y	+	>+++	+++	>+++	>++	>++++	N	N	N	N

Note: AmB, amphotericin B; Flu, fluconazole; N, not more resistant than parental strains. The number of ‘+’ indicates the level of tolerance compared to the parental strain. Each ‘+’ represents ~10X increase in survival after the stress challenge.

**Figure 5:**
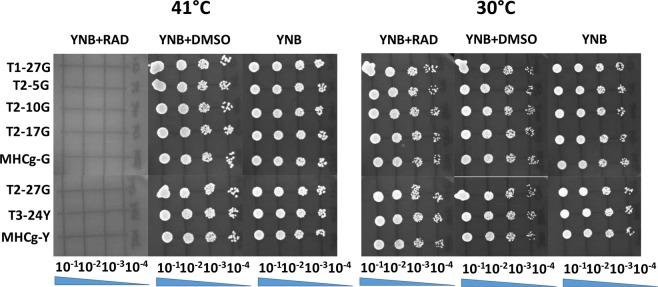
The effects of radicicol (RAD) on the thermotolerance of adaptive mutants and parental strains (MHCg-Y and MHCg-G). The cells were grown on YNB plates supplemented with 2% dextrose in the presence or absence of 5 µM of RAD at both 30 °C and 41 °C.

The heat shock proteins (HSP) play crucial roles for cell survival in the presence of environmental stressors including heat. Thus, the adaptive mutants were heat challenged at 41 °C in the presence of the HSP90 inhibitor radicicol to determine if HSP90 is essential for the improved hyperthermal tolerance observed. Presence of radicicol resulted in no growth inhibition in any strains tested at normal growth temperature (30 °C), but completely inhibited the growth of all strains at 41 °C (Fig. 5), demonstrating that HSP90 is required for thermo-tolerance in *C*. *glabrata* and that the improved adaptation to hyperthermal stress in the adaptive mutants is HSP90-dependent.

### Whole genome resequencing to identify potential beneficial mutations

All six isolated adaptive mutants and the parental strains MHCg-G and MHCg-Y were resequenced; and a total of 20 *de novo* mutations were identified from these adaptive mutants (Table S2). Among these mutations, 17 are in coding sequences and 2 are within 1 kbp of at least one coding sequence. Four mutations were identified in more than one adaptive mutant, three of which are present in two pseudogenes (CAGL0F00110g and CAGL0B05093g). One mutation in gene CAGL0B02739g (C766A) is present in all four adaptive mutants isolated from population T2. CAGL0B02739g encodes the homolog to the *S*. *cerevisiae* mitogen activated protein kinase kinase kinase (MAPKKK/MEKK) Ste11p; CgSte11 is reported to function in hypertonic stress response, filamentous growth, and virulence in *C*. *glabrata*^26^. Interestingly, mutations in *CgSTE11* were also identified in the two adaptive mutants sequenced from population T1 (C1661T) and T3 (C766T); the mutation in CgSte11 in strain T3-24Y from population T3 (C766T, Pro256Ser) occurred at the same nucleotide position as that identified in mutants from population T2 (C766A, Pro256Thr), but with a different amino acid change. We also performed population-level sequencing to identify all mutations present at a minimum of 10% frequency in the population over the course of the evolution (raw sequencing data in SRA with the accession number of SRP112804). The time-course population-level sequencing results from T1, T2, and T3 revealed that the frequency of *CgSTE11* mutations increased rapidly over time and fixed in all populations at the end of the evolution experiment, strongly suggesting that CgSte11 plays an important role in the adaptation of *C*. *glabrata* to heat stress (Fig. S5).

Gene Ontology (GO) analysis was performed using the ‘GO term Finder’ from the Candida Genome Database (http://www.candidagenome.org/) to identify any enriched biological processes or molecular functions among the genes with identified mutations in the coding regions or in potential regulatory regions in the mutants. The results revealed that genes functioning in the osmosensory signaling pathway (CAGL0H06567g, CAGL0B02739g [*CgSTE11*], and CAGL0M11748g) are significantly enriched with a p-value of ~0.0028, which suggests the involvement of the components of the osmosensory signaling pathway in *C*. *glabrata* adaptation to heat stress. Among these genes, CgSte11 has been shown to play a key role in the osmotic stress response in *C*. *glabrata*^26^. Since the majority of the identified mutations are in genes that have not been characterized in *C*. *glabrata*, we performed our analysis based on the functions of their *S*. *cerevisiae* orthologs (from Saccharomyces Genome Database and Candida Genome Database) to search for mutated genes that potentially contributed to the improved environmental stress tolerance in *C*. *glabrata*. In total, we found 9 candidate genes whose orthologs are known to impact *S*. *cerevisiae* tolerance to at least one of the stressors tested (Table S3). Surprisingly, 5 of the 9 genes (CAGL0C05599g, CAGL0D06732g, CAGL0F03311g, CAGL0G08602g, and CAGL0H06281g) contain likely inactivating mutations (either nonsense or frame shift mutations). Null mutations in four of these genes are known to impair tolerance to some environmental stressors (*e*.*g*. ethanol, H_2_O_2_, HCl) in *S*. *cerevisiae*. Assuming the functions are conserved between the *C*. *glabrata* genes and their *S*. *cerevisiae* orthologs, these mutant alleles (except for CAGL0C05599g) may potentially decrease stress tolerance in *C*. *glabrata*; surprisingly, the adaptive mutants harboring these mutations exhibit increased tolerance to many of the same stressors. In contrast, CAGL0C05599g is predicted to be an ortholog to the *S*. *cerevisiae LRG1* gene, which has been reported to increase thermal and acetic acid tolerance when nullified^27,28^. Thus, the nonsense mutation in CAGL0C05599g found in mutant T2-27G may be responsible for the improved tolerance to thermal and acetic acid stress in this mutant. In addition, mutations in 2 of the 9 genes (CAGL0M05709g and CAGL0M11748g) are missense mutations. The *S*. *cerevisiae* ortholog of CAGL0M05709g is *SGF73*, which has been reported to play a role in the resistance to H_2_O_2_ and ethanol^29,30^. *HOG1* is the *S*. *cerevisiae* ortholog of CAGL0M11748g, and is known to impact the resistance of yeast to boric acid and H_2_O_2_^29,31^.

### Phenotypic effects of reconstructed *CgSTE11* mutants on general stress tolerance

**Figure 6:**
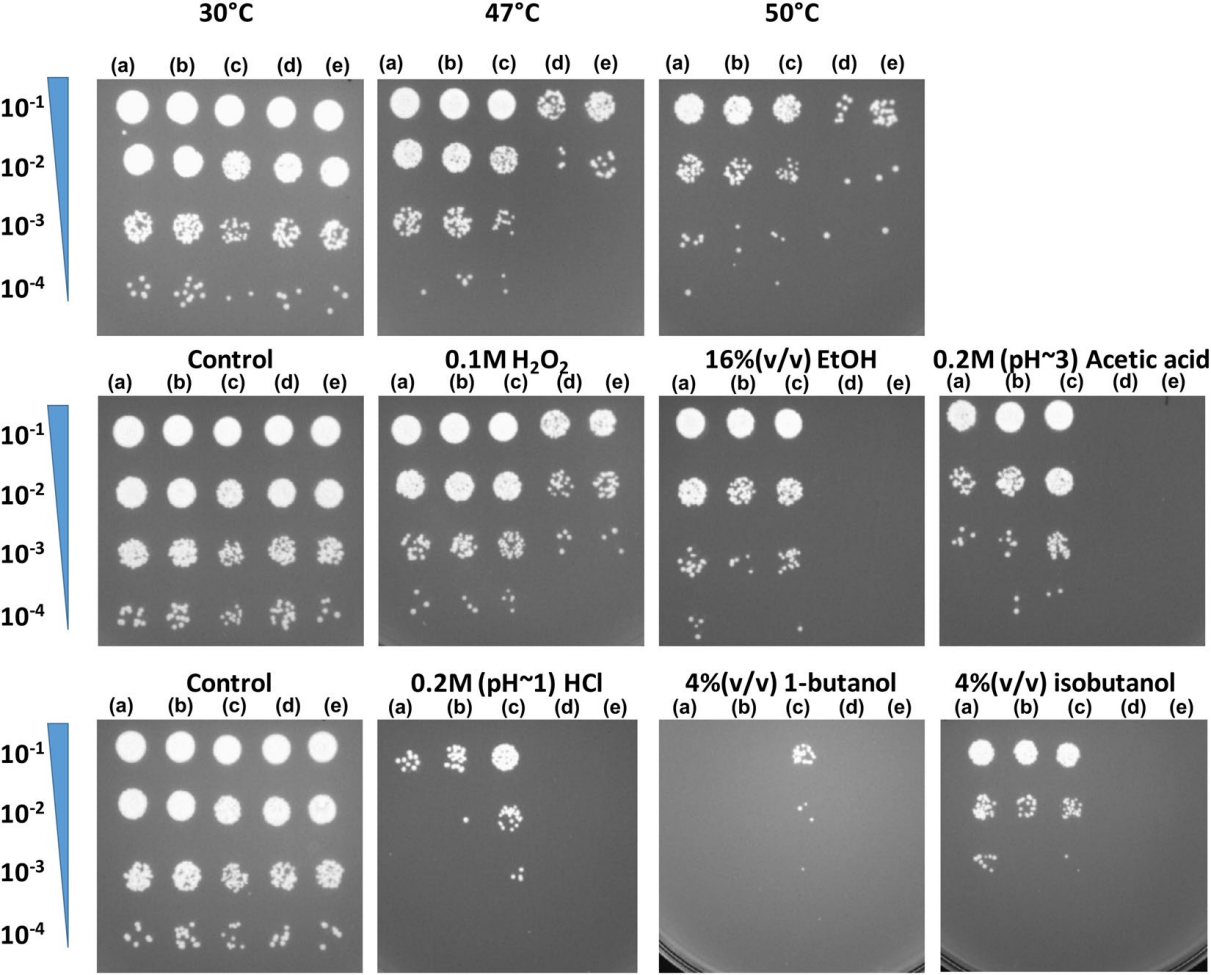
The stress tolerance for reconstructed *CgSTE11* mutants (sMH083 (**a**), sMH082 (**b**), sMH081 (**c**)) and the two controls, sMH080 (reconstructed wild-type; (**d**)) and ATCC 2001 (**e**) using 1-hr shock assays.

The fixation of mutations in *CgSTE11* (CAGL0B02739g) in all 3 parallel populations and the enriched osmosensory signaling pathway, which Ste11 in *S*. *cerevisiae* is a component of, from the GO analysis led us to speculate that the identified mutations in this gene are causative for the enhanced heat tolerance observed in the evolved mutants. Currently, genetic engineering tools are limited in *C*. *glabrata* compared with *S*. *cerevisiae*^32^. Thus, variants of the *CgSTE11* alleles were reconstructed in a wild-type background using a strategy based on the MoClo-derived assembly developed for *S*. *cerevisiae* (see Materials and Methods for details)^33,34^. Specifically, *CgSTE11* C1661T (identified in the T1 population), *CgSTE11* C766A (identified in the T2 population), and *CgSTE11* C766T (identified in the T3 population) alleles were reconstructed in the parental background (ATCC 2001) to generate strains sMH081, sMH082, and sMH083, respectively. After looping out the SAT1 cassette, a scar of ~109 bp remained between the coding sequence of *CgSTE11* and the 3′ UTR, which can potentially impact the expression of the gene^35^. Thus, a control strain sMH80 was generated via the same procedure to reintroduce the wild-type allele into ATCC 2001, to assess any phenotypic impacts due to the presence of the FRT scar. These strains (sMH080, sMH081, sMH082, and sMH083) along with ATCC 2001 were subjected to short-term challenge to environmental stressors, and the results are shown in Fig. 6. No significant differences in the tolerance to any stressors were observed between ATCC 2001 and control strain sMH080, suggesting that the FRT scar did not significantly impact the expression of the gene. All 3 *CgSTE11* variants acquired increased tolerance to several stressors tested, including heat, hydrogen peroxide, ethanol, acetic acid, *etc*., as compared to both wild-type controls. The results demonstrate that mutations in *CgSTE11* contributed to the enhanced tolerance to various environmental stressors observed in the adaptive mutants. Tolerance assay using a *ste11* deletion strain showed that *ste11∆* does not enhance hyperthermal tolerance in *C*. *glabrata* (see Supplementary Fig. S6), suggesting that the *CgSTE11* variants selected for are not inactivating mutations.

### Phenotypic effects of reconstructed *CgSTE11* mutants on biofilm formation

**Figure 7:**
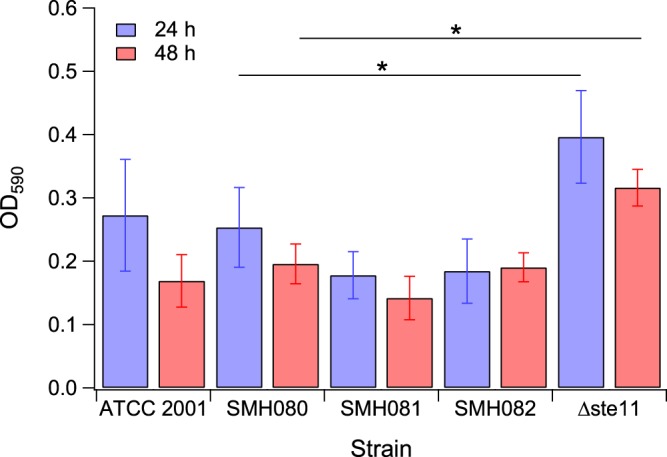
Biofilm formation of *CgSTE11* wild-type (ATCC 2001 and sMH080), reconstructed mutants (sMH081 and sMH082), and *ste11∆* strains. Blue bars: 24 hour biofilms. Red bars: 48 hour biofilms. Asterisks indicate p-values < 0.01 using 2-tailed Student t-test (unpaired with unequal variance).

In addition to high osmotic stress and cell wall stress response, MAPK signaling pathways have been shown to participate in adhesion^36,37^ and biofilm formation^38,39^ in *S*. *cerevisiae* and *C*. *albicans*. Expression of *ScSTE11* is downregulated in biofilm versus free planktonic cells^38^. In *C*. *albicans*, a *CaSTE11* mutant exhibited stronger biofilm formation compared with the wild-type^39^; interestingly, insertion mutants in *CaSTE11* showed significantly reduced adhesion compared with wild-type^37^. The impacts of two of the adaptive mutations in *CgSTE11* (strains sMH081 and sMH082) on biofilm formation was assessed using biofilm quantification assay with crystal violet staining. Control strains ATCC 2001 and sMH080 along with *ste11∆* were also analyzed. Results show no significant difference between biofilm formation in ATCC 2001 and sMH080 as expected (Fig. 7). The *ste11∆* strain exhibited higher biofilm formation, whereas strains sMH081 and sMH082 showed slight decrease in biofilm formation compared with the wild-type controls. The results showed that similar to *S*. *cerevisiae* and *C*. *albicans*, Ste11 also plays a role in biofilm formation in *C*. *glabrata*.

## Discussion

In this work, we identified and confirmed that mutations in *CgSTE11* found in hyperthermal evolved mutants contribute to enhanced tolerance to a wide range of environmental stressors and that the MAPK kinase kinase likely plays a key role in cellular response to environmental stressors in *C*. *glabrata*. CgSte11 is assumed to be a key component in the Sho1 branch of HOG signaling pathway based on its ability to partially complement the loss of Ste11 in *S*. *cerevisiae*^26^. In addition to osmotic stress, the HOG signaling pathway in *S*. *cerevisiae* is also activated in response to oxidative stress^40,41^, and exposure to acetic acid (through the Sln branch)^42^. It has been shown that the Sho1 branch of HOG signaling in *C*. *glabrata* influences cellular tolerance to heat and weak organic acids^21^. Thus, it is likely that CgSte11 functions in the modulation of heat and weak acid tolerance. In agreement with this theory, our experimental results showed that the heat tolerance conferring mutations in *CgSTE11* also significantly improved the tolerance of *C*. *glabrata* to acetic acid, suggesting that the mutated *CgSTE11* may improve stress tolerance, at least to heat and weak acid, by increasing the activity of HOG signaling in *C*. *glabrata*. It is important to note that strain ATCC 2001 used in this work has a truncated CgSsk2 allele, resulting in an inactive Sln1 branch of the HOG1 signaling pathway^21^. Thus, it is possible that the impact on weak acid tolerance conferred by the CgSte11 variants may be obviated in a strain with an active Sln1 branch. Further investigation in a different background will verify whether a bypass through this other HOG1 branch will impact the effects of the CgSte11 variants on organic acid tolerance observed in ATCC 2001. Studies have shown that CgSte11 is required for tolerance to hyperosmotic stress, nitrogen starvation induced filamentation, and virulence in *C*. *glabrata*^26^. Interestingly, we did not observe improved tolerance to common osmotic agents, including glucose and sorbitol, in isolated adaptive mutants with mutations in *CgSTE11*. In *S*. *cerevisiae*, the acetate activated Hog1 was found to not enhance hyperosmotic tolerance^42^, suggesting complex regulation of the activity of Hog1. Therefore, it may be that the CgSte11 variants did not lead to activation of Hog1, or activated Hog1 in a manner that did not impact the osmotolerance of *C*. *glabrata*. The exact mechanism remains to be investigated.

In response to various environmental stressors, cross-talk between different signaling cascades is necessary to coordinate the regulation of gene expression for cellular survival, especially when the stress level exceeds the capacity that can be mitigated by a single signaling cascade^43,44^. Much of our knowledge on fungal signaling cascades come from studies in *S*. *cerevisiae*. The cell wall integrity (CWI) pathway orchestrates cellular responses such as cell wall biosynthesis and actin organization necessary for the cell to withstand various stresses^45^. In *S*. *cerevisiae*, the CWI pathway is activated by heat stress^5^, involved in tolerance to low pH^46^, and increased activation of the CWI pathway has been shown to enhance tolerance to isobutanol^47^. Evidence have started to emerge showing that Ste11 plays a critical role in the cross-talk between the CWI and the HOG pathway in *S*. *cerevisiae* in response to environmental stressors, such as oxidative and thermal stress^13,48^. Leng and Song reported that Nst1p mediates the interaction between Ste11p and Mkk1/2p, connecting the HOG and pheromone signaling pathways to the CWI pathway in response to heat and pheromone^13^. In addition, Jin *et al*. showed that in *S*. *cerevisiae*, Ste11p may activate Mkk1/2p and subsequently Slt2p and Kdx1p, which results in the nuclear release and subsequent degradation of the repressor cyclin C in response to high levels of oxidative stress to activate stress-responsive gene expression^48^. Consistent with these reports, our data showed that mutations in *CgSTE11* conferred improved tolerance to both oxidative and thermal stress. Although differences exist, most of the components from the HOG and CWI pathway are conserved (at the sequence level) between *S*. *cerevisiae* and *C*. *glabrata*^18^. Given the high level of stressors used in this work, cross-talk between multiple signaling cascades, such as HOG and CWI pathways, may be involved in the ability of the isolated adaptive mutants to survive in these environmental stressors. Blast analysis of putative signaling proteins in the HOG and CWI pathways in *C*. *glabrata* (*e*.*g*. Ste11p [64% identity, 77% similarity], Mkk1p [58% identity, 67% similarity], Mkk2p [68% identity, 81% similarity], Nst1p [77% identity, 90% similarity], Slt2p [77% identity, 84% similarity]) showed that they have relatively high similarity and identity at the amino acid level to their putative *S*. *cerevisiae* orthologs. Further detailed studies are needed, however, to determine the extent of conservation between the MAPK signaling pathways between these two species.

In addition to the MAPK signaling pathways, heat shock proteins (HSP), which are involved in protein folding, prevention of protein aggregation, degradation and repair of damaged proteins, also play a crucial role for cell survival in environmental stressors. HSP90 family of HSPs is well-conserved among eukaryotic cells^49^. In *S*. *cerevisiae*, the HSP90 family has two isomers, encoded by *HSP82* and *HSC82*^50^. Although to different extents, the expression of both isomers of HSP90 are significantly induced in response to heat challenge in *S*. *cerevisiae*^50^. Acting as an evolutionary capacitor, HSP90 is able to mask phenotypic effects of pre-existing genetic variants that are not adaptive to keep them from being lost in the evolving populations and to release their phenotypic effects rapidly when its activity is compromised (*e*.*g*. by environmental stress)^51–54^, which facilitates evolutionary changes over long timescale. In addition, HSP90 has been proposed to function as a biological transistor that tunes cellular outputs (*e*.*g*. morphology, cell cycle progression, and drug resistance) to environmental inputs by regulating activities of core signaling pathways (*e*.*g*. CWI and Ca^2+^-calmodulin pathways)^49^. Members of the MAPK signaling pathways, such as Ste11p and Slt2p, are among the numerous proteins that interact with members of the HSP90 family^55,56^. Hsp90 is reported to impact the kinase activity of Ste11p in *S*. *cerevisiae*^55,57^. In addition, it has also been reported that appropriate post-translational modifications of HSP90 is necessary for efficient chaperoning of Ste11p and Slt2p^58^, suggesting that the chaperoning by HSP90 is important for Ste11p to function properly in signal transduction to regulate cellular response to environmental stressors. Indeed, our data showed that parental strains and isolated adaptive mutants with mutations in *CgSTE11* are hypersensitive to high temperature exposure (41 °C) in the presence of the HSP90 inhibitor radicicol. Taken together, the results suggest that the activity of the CgSte11 depends on the chaperoning of the HSP90 in *C*. *glabrata* in a similar fashion to *S*. *cerevisiae*.

In addition to mutations in *CgSTE11*, several other mutations were identified in the isolated adaptive mutants. Interestingly, in population T2, temporal isolates exhibited increased tolerance to some stressors (*e*.*g*. HCl, ethanol, H_2_O_2_) as a function of time. As mutations in *CgSTE11* are already present in the earliest isolate (T2-5G), it strongly suggests that additional mutations found in the later isolates within the T2 population further contribute to environmental stress tolerance of *C*. *glabrata* to various abiotic stresses.

## Conclusion

In this work, we identified significant cross-tolerance between heat and other environmental stressors such as oxidative, low pH, organic acid, and organic solvent in *C*. *glabrata* mutants evolved under periodic hyperthermal challenge. Focusing on the genotype-phenotype relationship in isolated adaptive clones, we demonstrated that mutations in *CgSTE11* is causative for the observed enhanced tolerance to heat and several other environmental stressors. Based on these results, we postulate that the identified mutations in *CgSTE11* likely led to enhanced activity of CgSte11, with impacts in multiple MAPK signal transduction cascades to enhance cellular resistance to several environmental stressors. Further experiments, especially detailed functional analysis of the mutated CgSte11 and components of the signaling pathways are needed to determine the exact molecular mechanism of how CgSte11 mediates tolerance to environmental stressors in *C*. *glabrata*. The work reported here shed light on the importance of the MAPK kinase kinase *STE11* in adaptation to environmental stressors in this important and yet under-studied organism.

## Materials and Methods

### Fungal strains and culture medium

Unless specified, Yeast Nitrogen Base (YNB) supplemented with 2% (w/v) dextrose is used as the media in all experiments. *C*. *glabrata* strains used in this study are derivatives of ATCC 2001. The primers, plasmids, and strains used in this study are listed in Supplementary Table S1.

The fluorescent *C*. *glabrata* strains, MHCg-Y and MHCg-G (as KKY and KKG, respectively, previously used in^34^), were constructed as follows: the gene encoding green fluorescent protein (GFP) was amplified from pGS62 plasmid^22^ using primer pair of HE1_F and HE1_R; the gene encoding yellow fluorescent protein (YFP) was amplified from pGS63^22^ using primer pair HE2_F and HE2_R. The PCR products and the vector yEPGAP-cherry^59^ were digested with EcoRI and XhoI. Each insert (GFP or YFP) was ligated with the vector backbone (replacing the yEMRFP gene on yEPGAP-cherry) to generate plasmids yEPGAP-GFP and yEPGAP-YFP, respectively. The SAT1 marker, amplified from yEP352-SAT1^60^ plasmid using the primers HE4_F and HE4_R, was assembled with the SalI-digested yEPGAP-GFP and yEPGAP-YFP via Gibson assembly^61^ to create plasmid yEPGAP-GFP-SAT1 and yEPGAP-YFP-SAT1, respectively. Gibson assembly reactions were carried out using Phusion® High-Fidelity DNA Polymerase (NEB), Taq DNA ligase (NEB), and T5 exonuclease (NEB); and the reaction was incubated at 50 °C for 1 hour. The 5′ and 3′ flanking regions for the fluorescent integration cassettes were amplified from genomic DNA (gDNA) of ATCC 2001 using the primer pairs HE6_F and HE6_R and HE7_F and HE7_R respectively, which contain homologous sequences to the pseudo gene CAGL0C01067g. The GFP and YFP cassettes were amplified from yEPGAP-GFP-SAT1 and yEPGAP-YFP-SAT1, respectively; and then assembled with the two flanking regions and BamHI-digested plasmid yEPGAP-cherry by Gibson assembly to create the fluorescent integration plasmids yMH-CgI-GFP and yMH-CgI-YFP. The fluorescent integration plasmids were then digested with SphI and integrated into the genome of ATCC 2001 by electroporation^62^ to generate the fluorescently marked strains MHCg-G and MHCg-Y, respectively.

The mutant alleles of CAGL0B02739g (CgSTE11) identified from the three evolved populations (T1, T2, and T3) were introduced into ATCC 2001 by first amplifying the partial coding sequences (~985 bp) containing the desired mutations in CAGL0B02739g from the gDNA of T1-27G, T2-5G, or T3-24Y with primer pair TE9_F and TE9_R to generate the 1^st^ part of the DNA fragment. The 2^nd^ part (~367 bp) was synthesized as a gBlock DNA fragments (Integrated DNA Technologies, Inc) to remove the existing BsaI site via a single nucleotide substitution without causing an amino acid change; and was joined with the 1^st^ part by overlapping PCR using the primer pair TE9_F and TE10_R. The partial coding sequence of the wild-type version of CAGL0B02739g was constructed in a similar way using the gDNA of ATCC 2001 as the template with the same primer pairs. The 3′ untranslated region (3′UTR) of CAGL0B02739g was amplified from the gDNA of ATCC 2001 with primers TE11_F and TE11_R. In addition, the SAT1-FLP cassette was amplified from the plasmid yEP352-SAT1 with primers TE12_F and TE12_R. These DNA fragments were each first cloned into the entry vector pYTK001 from the Yeast Tool Kit^33^ to create a set of part plasmids (pMH032, pMH033, pMH034, and pMH041, pMH042, and pMH043); and then were assembled with parts from pYTK002, pYTK067, and pYTK095 to construct four cassette plasmids pMH035, pMH036, pMH037, and pMH038 that contain the replacement cassettes for mutant alleles of CAGL0B02739g from populations T1, T2, T3 and the wild-type control, respectively. These plasmid constructions follow the MoClo-derived assembly described by Lee, *et al*.^33^. The part plasmids used in the construction of the replacement cassette plasmids were verified by Sanger sequencing. Each replacement cassette was digested from the cassette plasmids by BsmBI (Thermo scientific) and integrated into the genome of ATCC 2001 using the Easy Frozen-EZ Yeast Transformation II Kit (Zymo Research) following the manufacturer recommended protocol. Positive transformants were selected on nourseothricin (100 µg/mL) YPD plates. The SAT1-FLP cassette was looped out spontaneously by growing the transformants in YPD medium without nourseothricin and subsequently verified by both colony PCR using primers TE25_F and TE25_R and Sanger sequencing.

The *C*. *glabrata ste11∆* deletion strain (*ste11*::SAT1 *his3∆ leu2∆ trp1∆)* was a gift from Karl Kuchler at the Medical University Vienna. All strains used in experiments comparing the *ste11∆* strain were cultured in synthetic complete (SC) media.

### Adaptive evolution of *C*. *glabrata* to thermal stress

The adaptive laboratory evolution experiments were carried out in 25 ml cultures in 125 ml flasks at 30 °C. The starting populations consisted of approximately equal numbers of MHCg-G and MHCg-Y strains. Three populations (T1, T2, and T3) were evolved in parallel. Each population was serially transferred daily and periodically heat-challenged following a two-phase strategy (Fig. 1a). During Phase I (P1), 1 ml of cell culture after 24-hour growth was challenged with heat stress for 30 minutes, followed by inoculation into 24 ml of fresh growth medium for overnight recovery and growth. In Phase 2 (P2), 250 µl of the overnight culture from the P1 phase was transferred into 24.75 ml of fresh growth medium; and the cells were grown for 24 hours to allow further recovery from the heat challenge. The two-phase strategy was repeated until the end of the evolution. During the evolution, the OD_600_ of the populations were measured by sampling the culture before and after each transfer. Frozen stocks of the evolving populations sampled immediately before each stress challenge were prepared in 25% (v/v) glycerol and stored at −80 °C for future characterizations. When a significant increase in OD_600_ is observed after the second recovery phase, the thermotolerance of the evolving populations was measured using a modified method used for the general stress tolerance (described in the “Phenotypic characterizations of adaptive clones and population samples” section below) to confirm any improvements in hyperthermal tolerance. Briefly, the population samples were challenged with a temperature gradient starting from the current level used during evolution to 2 °C above (in 1 °C increment) for 30 minutes; the remaining steps were unchanged. The selective pressure (temperature) was ramped up to the new level that led to at least a 10-fold reduction in viability compared to the control (challenged at 30 °C). The relative proportions of the two subpopulations (MHCg-G and MHCg-Y) within each evolving population was measured using FACScan flow cytometer (BD Biosciences, San Jose, CA). The isolation of the adaptive mutant for a given population sample was performed as follows: a minimum of 8 colonies from a given population sample were randomly chosen and their relative thermotolerance were estimated using the general stress tolerance protocol described above; the isolate with the highest heat tolerance was selected as the adaptive mutant from that population. The naming of the adaptive mutants is based on the population samples from which they were isolated and the color of the fluorescent protein expressed (*e*.*g*. mutant T2-5G was isolated from the GFP subpopulation from time point 5 of population T2).

### Phenotypic characterizations of adaptive clones and population samples

#### Survival assay

The thermo-tolerance of population samples and isolated mutants were assessed after one-hour exposure to the temperature at which the samples were challenged at during the evolution experiment. Each sample was grown to an OD_600_ between 2.0~3.0 in YNB supplemented with 2% (w/v) dextrose at 30 °C, then the OD_600_ was normalized to ~0.5 before the heat challenge. 500 µl of the normalized samples were challenged with heat stress for 1 hour with shaking at 250 RPM in a shaking incubator; cells in the control group were incubated at 30 °C for 1 hour in a shaker. After the challenge, the cells (both control and heat-challenged groups) were placed on ice for 5 minutes, then subjected to serial 10-fold dilutions using culture medium; and the 10^−4^-fold diluted cells were plated on YNB plates and incubated at 30 °C for two days before counting the number of colony forming units (CFUs). The survival rate was calculated as the ratio of the CFUs of heat-challenged cells to CFUs of the control group. At least three biological replicates for each sample were used.

#### General stress tolerance

The stress tolerance of isolated adaptive mutants was characterized using the following steps: 1) cultures were grown to an OD_600_ between 2.0~3.0 in YNB at 30 °C, 2) the OD_600_ was normalized to ~0.5 for heat and ~1.0 for other stresses before the challenge, and 3) 500 µl of the normalized samples were then challenged by the stressor-of-interest for 1 hour in a shaker. For the control group, cells were incubated in the absence of stressor for 1 hour at 30 °C in a shaker. The stressors used include 4% (v/v) isobutanol, 4% (v/v) 1-butanol, 16% (v/v) ethanol, 0.2 M of HCl (pH~1.0), 0.2 M of acetic acid (pH~3.0), 100 mM of H_2_O_2_, and heat at 47 °C and 50 °C. For the stressors other than heat, the cells (both control and stress-challenged group) were pelleted after the challenge to remove the supernatant and then serially diluted (10-fold dilutions) using culture medium. For heat stress, the cells (both control and heat-shocked groups) were placed on ice for 5 minutes after the challenge before subjected to serial 10-fold dilutions using YNB. 5 µl of diluted cells (10^−1^, 10^−2^, 10^−3^, and 10^−4^) were spotted on YNB agar medium supplemented with 2% dextrose and incubated at 30 °C for two days and photographed for analysis.

We also evaluated the tolerance of adaptive mutants to osmotic stressors (glucose and sorbitol) and antibiotics (amphotericin B and fluconazole) in YNB medium. For osmotic stressors, we cultured 200 µl of cells with OD_600_ normalized to ~0.02 in YNB medium in the presence of a concentration gradient of glucose (0%, and 20% to 35% w/v with 5% increment) and sorbitol (0 M, and 1.5 M to 3 M with 0.5 M increment) in 96-well plates at 30 °C and 200 rpm. The OD_600_ was measured using a microplate reader (Tecan Group Ltd., Switzerland). Growth curve fitting of OD_600_ data with the late-stationary-phase time points excluded was performed using DMFit (www.ifr.ac.uk/safety/DMfit) to measure specific growth rates using the model of Baranyi and Roberts^63^. For antibiotics, a modified test based on the CLSI broth microdilution method^64^ was performed via the following steps: (1) cells were grown on SDA plates (1% Peptone, 4% Dextrose, 1.5% agar, adjusted to pH 5.6 with HCl) at 35 °C for 24 hours, (2) 4 or 5 colonies ~1 mm in diameter were picked from the SDA plates and suspended in 1 ml of sterile 0.85% saline (8.5 g/L NaCl in water), (3) the OD_600_ was normalized to ~0.5 and the normalized cultures were diluted 50 fold first using 0.85% saline, followed by 50-fold and 5-fold dilution using YNB medium, (4) 100 µl of the diluted cultures were then mixed with 100 µl of YNB medium containing a concentration gradient of amphotericin B (0, and 0.25 µg/mL to 32 µg/mL in 2-fold serial dilution) or fluconazole (0, 1 µg/mL to 128 µg/mL in 2-fold serial dilution) in 96-well plate, 5) the 96-well plate were incubated at 35 °C without agitation for 48 hours in the dark, and 6) the OD_492_ of cell contents in each well of the 96-well plate was measured to estimate the biomass. The minimal inhibitory concentration (MIC) of antibiotics which result in at least 50% reduction in the biomass as compared to control (0 µg/mL) was determined using two biological replicates.

#### Radicicol treatment

Radicicol functions as an inhibitor of Hsp90^65^ and was used to assess whether the hyperthermal tolerance in the isolated mutants is Hsp90-dependent. Radicicol (Cayman chemical) was dissolved in DMSO to make the stock solution (2 mg/ml). The impact of radicicol on the hyperthermal tolerance of each strain was determined by normalizing overnight cultures of each strain (OD_600_ between 2.0~3.0) grown in YNB at 30 °C to an OD_600_ of ~0.5. The normalized samples were serially diluted in YNB, and 5 µl of diluted cells (10^−1^, 10^−2^, 10^−3^, and 10^−4^) were spotted on YNB agar medium supplemented with 2% (w/v) dextrose and 5 µM of radicicol. A control group spotted on YNB agar or YNB agar with or without 0.09% (v/v) DMSO in the absence of radicicol were included. Each set of samples were incubated at 30 °C and 41 °C for two days and photographed for analysis.

#### Biofilm quantification assay

Single colonies of *C*. *glabrata* strains (ATCC 2001, sMH080, sMH081, sMH082, *ste11∆*) were picked from agar plates and cultured in YPD broth at 37 °C. Overnight cultures were washed twice with PBS and resuspended in fresh YPD medium to a concentration of 2.5 × 10^7^/ml using a hemocytometer. 100 µl of cell suspension was added to each well of 96-well plates precoated with type I collagen (Corning BioCoat, Fisher Scientific). The cells were cultured for 24 h or 48 h at 37 °C without agitation for biofilm formation. Unattached cells and media were removed by inverting the plates, and tapping the plates gently on paper towels twice. Following a similar procedure, the established biofilms were washed twice with 200 µl PBS, then air dried. Once completely dried, 200 µL of 0.1% crystal violet was added to each well and incubated at room temperature for 20 minutes^66^. The plates were then submerged in a tray of DI water, inverted to remove water, and tapped two times to remove excess crystal violet stain from the wells and biofilms. 150 µL of 33% acetic acid was added to each well, and absorbance was measured at 590 nm in a plate reader (Neo2S Biotek). Three biological replicates with three technical replicates each were used for analysis.

### Next-Gen sequencing

The adaptive clones, T1-27G, T2-5G, T2-10G, T2-17G, T2-27G, and T3-24Y, from population T1, T2, and T3 and the two unevolved parental strains, MHCg-Y and MHCg-G were sequenced to identify the genetic mutations underlying the observed hyperthermal tolerance. In addition, temporal population samples from T1, T2, and T3 were sequenced as well in order to discover rare adaptive mutations (≥10%) and evaluate the temporal change in the population frequency of mutations. Genomic DNA was extracted using the YeaStar^TM^ Genomic DNA kit (Zymo Research). Genomic library preparation and sequencing were performed by the Texas A&M Genomics Center for sequencing on the Illumina HiSeq. 2500 platform using 100-bp pair-end reads. An average coverage of more than 32-fold was obtained for each clone, and ~200-fold for population samples. The raw sequencing data of these samples were deposited in SRA database (https://www.ncbi.nlm.nih.gov/sra) with accession number SRP112804. Reads were assembled against the CBS138 (ATCC 2001) reference genome, and each mutant genome was compared to the parental sequences to identify any *de novo* mutations using the CLC Genomics workbench (version 6.0.1). For calling of variants, the frequency threshold was set to be 90% for clones and 10% for population samples, and the forward/reverse balance was set at ≥0.3 for both. Otherwise, the default setting was used for the CLC Genomics workbench. Selected mutations were verified with Sanger sequencing and analyzed with Unipro UGENE.

### Bioinformatics analysis

The Gene Ontology (GO) analysis tool from Candida Genome Database (CGD)^67^ was used to identify any enriched biological processes and functional categories in the mutated genes. Since a large fraction (95.67%) of the genes in *C*. *glabrata* have not been characterized in detail^67^, functions of genes-of-interest were inferred based on their *S*. *cerevisiae* orthologs using available information from the Saccharomyces Genome Database (SGD)^68^.

## Supplementary information

Supplementary Information

## Data Availability

The datasets of genome sequencing generated during the current study are available in the SRA database with accession number SRP112804, https://www.ncbi.nlm.nih.gov/sra. All other data generated or analyzed during this study are included in this published article (and its Supplementary Information Files).
