# Supplementary material for: CgSTE11 mediates cross tolerance to multiple environmental stressors in Candida glabrata
Source: Sci Rep. 2019 Nov 19;9:17036. doi: 10.1038/s41598-019-53593-5 (PMC6863853; doi:10.1038/s41598-019-53593-5)
Supplement: Supplementary file 1 — Supplementary Information [file 41598_2019_53593_MOESM1_ESM.docx]

**Supplementary Information**

Title: *CgSTE11* mediates cross tolerance to multiple environmental stressors in *Candida glabrata*

Mian Huang^1^, Jibran Khan^2^, Manpreet Kaur^3^, Julian Daniel Torres Vanega^4&^, Orlando Andres Aguilar Patiño^4&^, Anand K. Ramasubramanian^3^, Katy C. Kao^3*^

^1^ Artie McFerrin Department of Chemical Engineering, Texas A&M University, College Station, Texas 77843, United States of America

^2^ Department of Biology, Texas A&M University, College Station, Texas 77843, United States of America

^3^ Department of Chemical and Materials Engineering, San José State University, San José, CA 95192, United State of America

^4^ Department of Chemical Engineering, Industrial University of Santander, Bucaramanga, Colombia

*Corresponding author

Email: kao.katy@gmail.com

**Supplementary Table S1-a.** List of primers used in the study

| Primer ID | Primer sequence (5'-->3') | Description |
| --- | --- | --- |
| HE1_F | CCCCCGAATTCATGAGTAAAGGAGAAGAACTT | Forward primer for amplification of GFP from pGS62 |
| HE1_R | CCCCCTCGAGTTATTTGTATAGTTCATCCATG | Reverse primer for amplification of GFP from pGS62 |
| HE2_F | CCCCCGAATTCATGAGTAAAGGAGAAGAACTT | Forward primer for amplification of YFP from pKKGS5 plasmid |
| HE2_R | CCCCCTCGAGCTATTTGTATAGTTCATCCATG | Reverse primer for amplification of YFP from pKKGS5 plasmid |
| HE3_F | GGCCAACACTTGTCACTACTTTCTC | Forward primer for verification of the replaced GFP and YFP in yEPGAP-GFP and yEPGAP-YFP |
| HE3_R | TCTTTCGAAAGGGCAGATTGTGT | Reverse primer for verification of the replaced GFP and YFP in yEPGAP-GFP and yEPGAP-YFP |
| HE4_F | gtaccgctcgagcagctgtgattgattgagGAGCGTCAAAACTAGAGAAT | Forward primer for amplification of SAT1 cassettes from yEP352-SAT1 plasmid |
| HE4_R | acttaagccttggcaacgtgttcaaccaagACTTCACATATGTTAGGCGT | Reverse primer for amplification of SAT1 cassettes from yEP352-SAT1 plasmid |
| HE5_F | taaaccattgagtgtCCGTTAGAATCATTTTGAAT | Forward primer for amplification of the TDH3-fluorescent cassette-SAT1 |
| HE5_R | ctcagtaactgtgctACTTCACATATGTTAGGCGT | Reverse primer for amplification of the TDH3-fluorescent cassette-SAT1 |
| HE6_F | ctccccgcgcgttggccgattcattaatcg*GCATGC*TCTCTGTGGTAGCAGATTAT | Forward primer for amplification of 5 prime flanking region from the pseudogene CAGL0C01067g |
| HE6_R | aaatgattctaacggACACTCAATGGTTTAGTAGC | Reverse primer for amplification of 5 prime flanking region from the pseudogene CAGL0C01067g |
| HE7_F | taacatatgtgaagtAACGGAAGTGTCCAGTGAAT | Forward primer for amplification of 3 prime flanking region from the pseudogene CAGL0C01067g |
| HE7_R | cggtgcgggcctcttcgctattacgccagg*GCATGC*CGTAGCACCTTGCATGTTAT | Reverse primer for amplification of 3 prime flanking region from the pseudogene CAGL0C01067g |
| HE8_F | CTTTACCATCTGGAACAAAG | Forward primer for verification of the integration of the YFP/GFP fluoresecnt cassette into the KK-CA-24 |
| HE8_R | ATATATTCTGTGTAACCCGC | Reverse primer for verification of the integration of the YFP/GFP fluoresecnt cassette into the KK-CA-24 |
| TE9_F | GCATCGTCTCATCGGTCTCAAACGAGGAAGGTAAGGATAAACTACGG | Forward primer for amplication of partial coding sequence of CAGL0B02739g variants from mutants |
| TE9_R | TGATTCTCCTGGTTTAGTGGTGA | Reverse primer for amplication of partial coding sequence of CAGL0B02739g variants from mutants |
| TE10_R | ATGCCGTCTCAGGTCTCACATACTATATGATATGACTCTCGAGCC | Reverse primer for amplification of the synthetic partial CAGL0B02739g CDS |
| TE11_F | GCATCGTCTCATCGGTCTCAATCCGATGAATATATTAACGAGAATG | Forward primer for amplication of 3 prime UTR of CAGL0B02739g |

**Supplementary Table S1-a.** Continued

| Primer ID | Primer sequence (5'-->3') | Description |
| --- | --- | --- |
| TE11_R | ATGCCGTCTCAGGTCTCACAGCACTTTGAAATACCAGACCG | Reverse primer for amplication of 3 prime UTR of CAGL0B02739g |
| TE12_F | GCATCGTCTCATCGGTCTCATATGTGACCATGATTACGAATTCGAGC | Forward primer for amplification of the SAT1-FLP cassette from yEP352-SAT1 plasmid |
| TE12_R | ATGCCGTCTCAGGTCTCAGGATGCTCTAGAACTAGTGGATCTG | Reverse primer for amplification of the SAT1-FLP cassette from yEP352-SAT1 plasmid |
| TE13 | CCTCTGACTTGAGCGTCG | sequencing primer for 3UTR of CgCAGL0B02739g (type 4) and partial CAGL0B02739g (type2) inserted in pYTK001 plasmid |
| TE14 | AATGCCTGCATCTGTGAG | sequencing primer for partial CAGL0B02739g (type2) inserted in pYTK001 plasmid |
| TE15 | GGGTTGTGTCAAGATCAC | sequencing primer for partial CAGL0B02739g (type2) inserted in pYTK001 plasmid |
| TE16 | CCAATCTTTGAATTATTC | sequencing primer for SAT1-FLP cassette (type4) inserted pYTK001 plasmid |
| TE17 | CCACGGTTAATAACATCC | sequencing primer for SAT1-FLP cassette (type4) inserted pYTK001 plasmid |
| TE18 | ACAACATTAGTCAACTCC | sequencing primer for SAT1-FLP cassette (type4) inserted pYTK001 plasmid |
| TE19 | AGGAATTCTGAACCAGTC | sequencing primer for SAT1-FLP cassette (type4) inserted pYTK001 plasmid |
| TE20 | TGGTGAGAACAGCGACCG | sequencing primer for SAT1-FLP cassette (type4) inserted pYTK001 plasmid |
| TE21 | TGTTGTGGGTGTGTGCTA | sequencing primer for SAT1-FLP cassette (type4) inserted pYTK001 plasmid |
| TE22 | GCCATAGCAAGCAGAGTC | sequencing primer for SAT1-FLP cassette (type4) inserted pYTK001 plasmid |
| TE23 | ATAGAAGAAGTTGCTGCT | sequencing primer for SAT1-FLP cassette (type4) inserted pYTK001 plasmid |
| TE24_F | GCAAACTACTCAAGAATCCTCCG | Forward primer for amplification of CAGL0B02739g variants |
| TE24_R | CCCAGGATGGTATCTCCGGT | Reverse primer for amplification of CAGL0B02739g variants |
| TE25_F | AGATACCATCCTGGGCCTCT | Forward primer for verification of the loopout of the SAT1-FLP marker in reconstructed strains with CAGL0B02739g variants |
| TE25_R | GGCATGTTTGGATCATACGCC | Reverse primer for verification of the loopout of the SAT1-FLP marker in reconstructed strains with CAGL0B02739g variants |
| TE26 | ACTATTCAAAGAACGTGC | primer for sequencing of the CAGL0B02739g variants |
| TE27 | AATGCCTGCATCTGTGAG | primer for sequencing of the CAGL0B02739g variants |

**Supplementary Table S1-b.** List of strains used in this study

| Strain ID | Genotype | Source |
| --- | --- | --- |
| ATCC2001 | N/A | ATCC |
| MHCg-Y | ATCC2001 CAGL0C01067g::ScTDH3p-YFP-SAT1 | this study |
| MHCg-G | ATCC2001 CAGL0C01067g::ScTDH3p-GFP-SAT1 | this study |
| T1-27G | evolved mutants | this study |
| T2-5G | evolved mutants | this study |
| T2-10G | evolved mutants | this study |
| T2-17G | evolved mutants | this study |
| T2-27G | evolved mutants | this study |
| T3-24Y | evolved mutants | this study |
| sMH080 | ATCC2001 ste11Δ::STE11-FRT | this study |
| sMH081 | ATCC2001 ste11Δ::STE11(C1661T)-FRT | this study |
| sMH082 | ATCC2001 ste11Δ::STE11(C766A)-FRT | this study |
| sMH083 | ATCC2001 ste11Δ::STE11(C766T)-FRT | this study |

**Supplementary Table S1-c.** The list of the plasmids used in this study

| Plasmid ID | Description | Markers | Source |
| --- | --- | --- | --- |
| yEPGAP-cherry | yEMRFP | URA3 AmpR | Keppler-Ross S, et al., 2008 |
| yEPGAP-GFP | GFP | URA3 AmpR | This work |
| yEPGAP-YFP | YFP | URA3 AmpR | This work |
| pGS62 | source of green fluorescent gene | AmpR | Kao KC and Sherlock G, 2008 |
| pGS63 | source of yellow fluorescent gene | AmpR | Kao KC and Sherlock G, 2008 |
| yEP352-SAT1 | source of the SAT1-FLP marker | URA3 AmpR SAT1 | Krauke Y and Sychrova H, 2011 |
| yEPGAP-GFP-SAT1 | GFP SAT1-FLP | URA3 AmpR SAT1 | This work |
| yEPGAP-YFP-SAT1 | YFP SAT1-FLP | URA3 AmpR SAT1 | This work |
| yMH-CgI-GFP | for integration of green fluorescent cassette at CAGL0C01067g | URA3 AmpR SAT1 | This work |
| yMH-CgI-YFP | for integration of yellow fluorescent cassette at CAGL0C01067g | URA3 AmpR SAT1 | This work |
| pYTK001 | Part Plasmid Entry Vector | GFP CmR | Lee ME, et al., 2015 |
| pYTK002 | ConLS | CmR | Lee ME, et al., 2015 |
| pYTK067 | ConR1 | CmR | Lee ME, et al., 2015 |
| pYTK095 | AmpR-ColE1 | AmpR | Lee ME, et al., 2015 |
| pMH032 | type 2 plasmid containing partial coding sequence of STE11 | CmR | This work |
| pMH033 | type 3 plasmid containing SAT1-FLP | CmR SAT1-FLP | This work |
| pMH034 | type 4 plasmid containing 3 prime UTR of STE11 | CmR | This work |
| pMH035 | cassette plasmid for replacement of STE11 with STE11-SAT1-FLP | AmpR SAT1-FLP | This work |
| pMH036 | cassette plasmid for replacement of STE11 with STE11(C1661T)-SAT1-FLP | AmpR SAT1-FLP | This work |
| pMH037 | cassette plasmid for replacement of STE11 with STE11(C766A)-SAT1-FLP | AmpR SAT1-FLP | This work |
| pMH038 | cassette plasmid for replacement of STE11 with STE11(C766T)-SAT1-FLP | AmpR SAT1-FLP | This work |
| pMH041 | type 2 plasmid containing partial coding sequence of STE11(C1661T) | CmR | This work |
| pMH042 | type 2 plasmid containing partial coding sequence of STE11(C766A) | CmR | This work |
| pMH043 | type 2 plasmid containing partial coding sequence of STE11(C766T) | CmR | This work |

**Supplementary Table S2.** Mutations identified in adaptive mutants isolated from population T1, T2, and T3 from next-generation sequencing analysis

| **SID** | **Gene^a^** | **Amino acid change** | **Non-synonymous** | **nearby gene ID^b^** |
| --- | --- | --- | --- | --- |
| T1-27G | CAGL0M11748g | Met188Ile | Yes | NA |
| T3-24Y | NA | NA | - | CAGL0L09955g(WU); CAGL0L09933g(WD); |
| T3-24Y | CAGL0G08602g | Pro732fs | Yes | NA |
| T2-10G | NA | NA | - | CAGL0I07843g(CD); CAGL0I07865g(WU); |
| T1-27G | CAGL0D06732g-T | Phe623fs | Yes | NA |
| T2-17G | CAGL0H06567g | Pro544Gln | Yes | NA |
| T3-24Y | CAGL0H06281g | Lys244fs | Yes | NA |
| T1-27G | CAGL0M05709g | Gln185Pro | Yes | NA |
| T2-27G | CAGL0C05599g | Glu531* | Yes | NA |
| T3-24Y | CAGL0B05093g-T, CAGL0B05148g | [Ser992Asn] | Yes | NA |
| T2-5G | CAGL0B05093g-T | Pro559fs | Yes | NA |
| T2-17G | CAGL0B05093g-T | Pro559fs | Yes | NA |
| T2-27G | CAGL0B05093g-T | Pro559fs | Yes | NA |
| T2-10G | CAGL0B05093g-T | *260Tyr | Yes | NA |
| T2-27G | CAGL0F03311g | Lys683* | Yes | NA |
| T2-5G | CAGL0B02739g | Pro256Thr | Yes | NA |
| T2-10G | CAGL0B02739g | Pro256Thr | Yes | NA |
| T2-17G | CAGL0B02739g | Pro256Thr | Yes | NA |
| T2-27G | CAGL0B02739g | Pro256Thr | Yes | NA |
| T3-24Y | CAGL0B02739g | Pro256Ser | Yes | NA |
| T1-27G | CAGL0B02739g | Thr554Ile | Yes | NA |
| T2-17G | NA | NA | - | NA |
| T2-5G | CAGL0C01067g-T, CAGL0C01078g | [Ala5fs] | Yes | NA |
| T1-27G | CAGL0F00110g-T | Leu763Gln | Yes | NA |
| T2-10G | CAGL0F00110g-T | Leu763Gln | Yes | NA |
| T2-27G | CAGL0F00110g-T | Leu763Gln | Yes | NA |
| T3-24Y | CAGL0F00110g-T | Leu763Gln | Yes | NA |
| T1-27G | CAGL0F00110g-T | Ser765Ser | No | NA |
| T2-10G | CAGL0F00110g-T | Ser765Ser | No | NA |
| T2-17G | CAGL0F00110g-T | Ser765Ser | No | NA |
| T2-27G | CAGL0F00110g-T | Ser765Ser | No | NA |
| T3-24Y | CAGL0F00110g-T | Ser765Ser | No | NA |
| a. the '-T' following the name of a gene indicates that the gene is a pseudogene; the '*' indicates a nonsense mutation;  b. the letters in the parenthesis following the gene ID describe the positions of identified mutations relative to the affected genes: W/C indicates the affected gene is in Watson strand/Crick strand; U/D indicates the mutations is in the upstream/downstream of the affected gene. For example, WU indicates that the affected gene is on Watson strand and the mutation is in its upstream. | | | | |
|  |  |  |  |  |
|  | | | | |

**Supplementary Table S3.** List of genes mutated in *C. glabrata* adaptive mutants whose orthologs in *S. cerevisiae* are known to impact environmental stress tolerance when nullified

| **Cg_Gene** | **Sc_Ortholog** | **Phenotype** |
| --- | --- | --- |
| CAGL0B02739g | STE11 | NA |
| CAGL0B05093g | NA | NA |
| CAGL0C05599g | LRG1 | increased tolerance to heat and acetic acid when nullified; "Jarolim S, et al. (2013)"; "Ding J, et al. (2013)" |
| CAGL0D06732g | FLO1 | decreased tolerance to ethanol when nullified; "Smukalla S, et al. (2008)"; |
| CAGL0F03311g | KSP1 | decreased tolerance to H2O2 when nullified; "Brown JA, et al. (2006)"; |
| CAGL0G08602g | RPI1 | decreased tolerance to propionic acid, heat, and H2O2 when nullified; "Mira NP, et al. (2009)"; "Jarolim S, et al. (2013)"; "Brown JA, et al. (2006)" |
| CAGL0H06281g | TPD3 | decreased tolerance to HCl, H2O2, acetic acid, and ethanol when nullified; "Banuelos MG, et al. (2010) "; "Outten CE, et al. (2005) "; "Kawahata M, et al. (2006) "; "Auesukaree C, et al. (2009) " |
| CAGL0H06567g | SLN1 | NA |
| CAGL0I07843g | ADH1 | increased tolerance to heat when nullified; "Jarolim S, et al. (2013)" |
| CAGL0I07865g | PHM7 | NA |
| CAGL0L09933g | CUE5 | NA |
| CAGL0L09955g | WHI2 | decreased tolerance to propionic acid, heat, H2O2, and ethanol when nullified; "Mira NP, et al. (2009)"; "Jarolim S, et al. (2013)"; "Brown JA, et al. (2006)"; "Teixeira MC, et al. (2009)" |
| CAGL0M05709g | SGF73 | decreased tolerance to H2O2 and ethanol when nullified; "Brown JA, et al. (2006)"; "Teixeira MC, et al. (2009)" |
| CAGL0M11748g | HOG1 | decreased tolerance to boric acid and H2O2 when nullified; "Schmidt M, et al. (2012)"; "Pascual-Ahuir A and Proft M (2007)"; "Brown JA, et al. (2006)" |
| CAGL0B05148g | NA | NA |
| CAGL0C01067g | NA | NA |
| CAGL0C01078g | NA | NA |
| CAGL0F00110g | NA | NA |

NA indicates not found


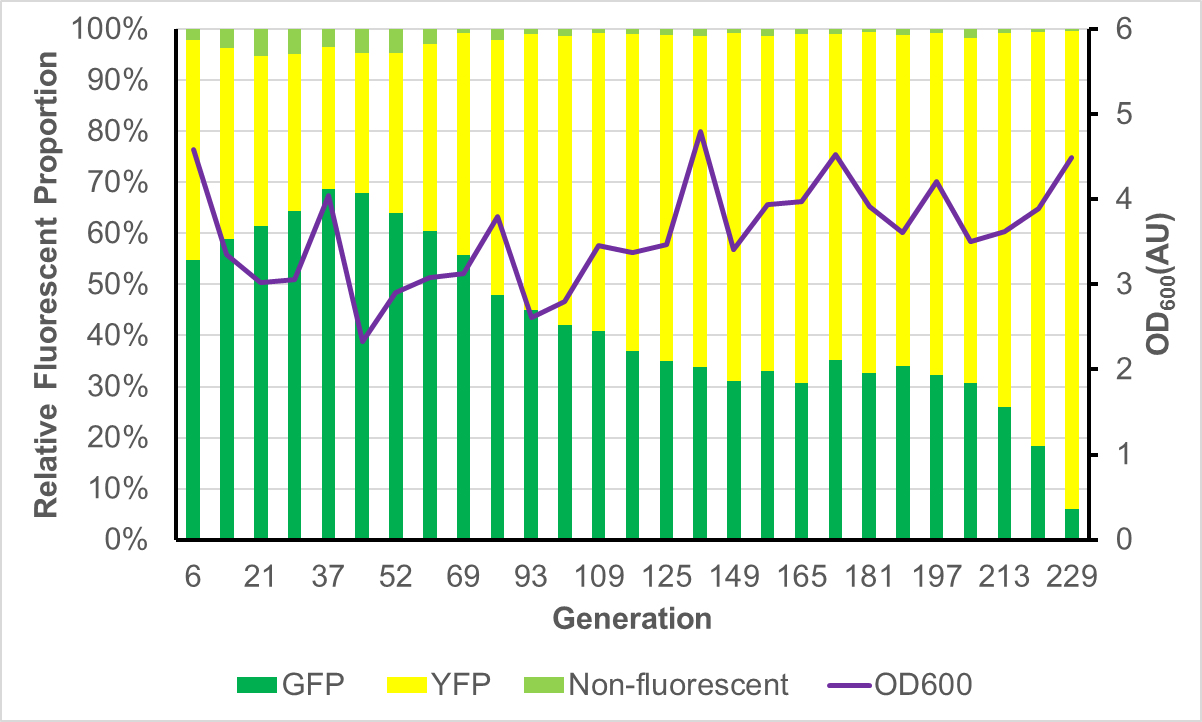


**Supplementary Figure S1.** Population dynamics of TC during adaptive evolution to hyperthermal stress. The colored bars represent the relative fluorescent proportions of green fluorescent subpopulation (dark green), yellow fluorescent subpopulation (yellow), and non-fluorescent subpopulation (light green) and the purple line represents the optical densities of the populations measured at 600 nm. The temperatures used in the 30-minute-heat-shock treatment during the evolution was 30°C. Note: During adaptive laboratory evolution, if beneficial mutants with enhanced tolerance to the imposed selective pressure are present in the population, an increase in final cell density may be expected. However, depending on the relative fraction of the adaptive mutants in the population, a significant increase in cell density may not be observed. On the other hand, having different colored cells in the population increases the “resolution” within the population by dividing it into trackable subgroups. Thus, tracking the relative proportion of fluorescently marked subpopulations can be more sensitive at detecting smaller changes in relative frequency of subpopulations.


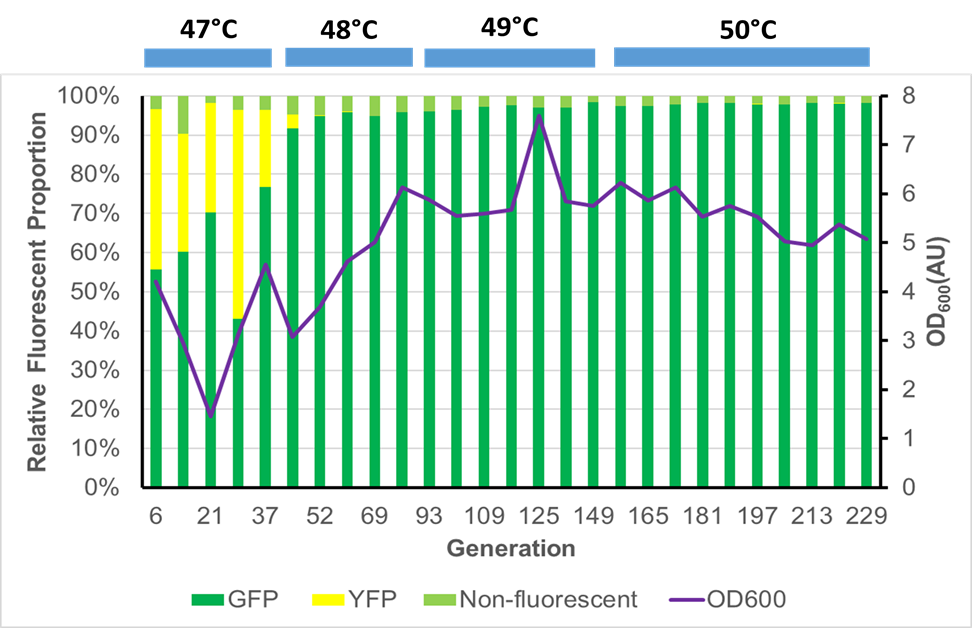


**Supplementary Figure S2.** Population dynamics of T1 during adaptive evolution to hyperthermal stress. The colored bars represent the relative fluorescent proportions of green fluorescent subpopulation (dark green), yellow fluorescent subpopulation (yellow), and non-fluorescent subpopulation (light green) and the purple line represents the optical densities of the populations measured at 600 nm. The temperatures used in the 30-minute-heat-shock treatment during the evolution were shown on the top.

**
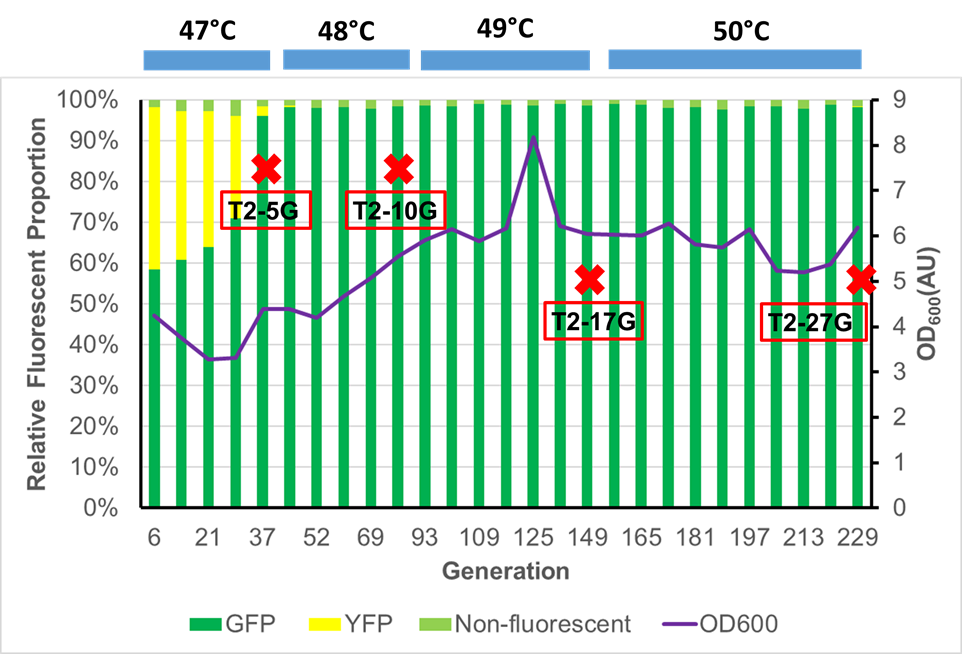
**

**Supplementary Figure S3.** Population dynamics of T2 during adaptive evolution to hyperthermal stress. The colored bars represent the relative fluorescent proportions of green fluorescent subpopulation (dark green), yellow fluorescent subpopulation (yellow), and non-fluorescent subpopulation (light green) and the purple line represents the optical densities of the populations measured at 600 nm. The temperatures used in the 30-minute-heat-shock treatment during the evolution were shown on the top. The isolated adaptive mutants were highlighted in red box; and the red crosses indicated the time points when they were isolated and the subpopulations from which they were isolated.


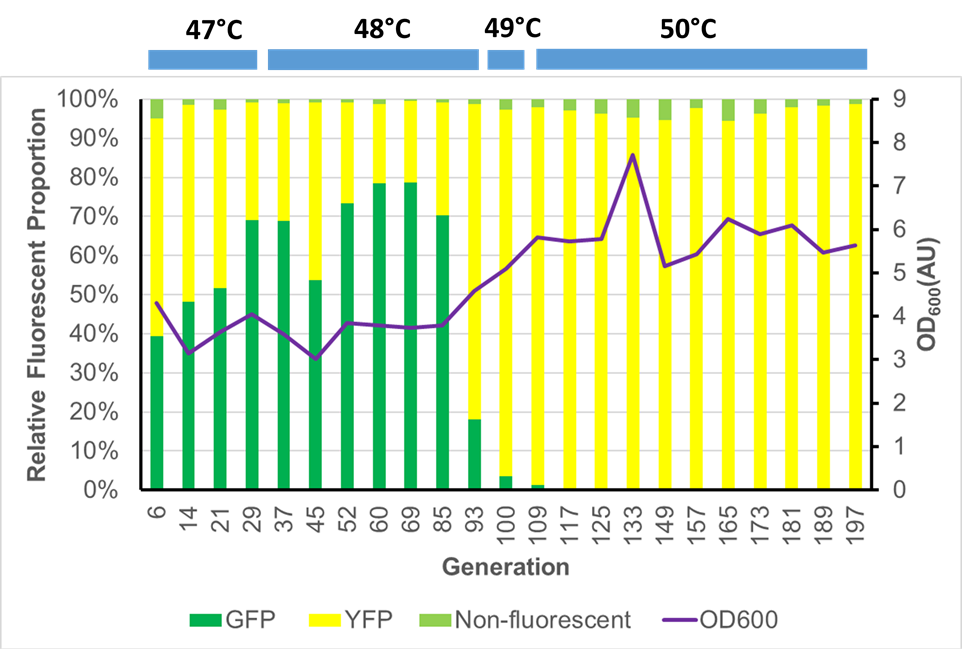


**Supplementary Figure S4.** Population dynamics of T3 during adaptive evolution to hyperthermal stress. The colored bars represent the relative fluorescent proportions of green fluorescent subpopulation (dark green), yellow fluorescent subpopulation (yellow), and non-fluorescent subpopulation (light green) and the purple line represents the optical densities of the populations measured at 600 nm. The temperatures used in the 30-minute-heat-shock treatment during the evolution were shown on the top.

**
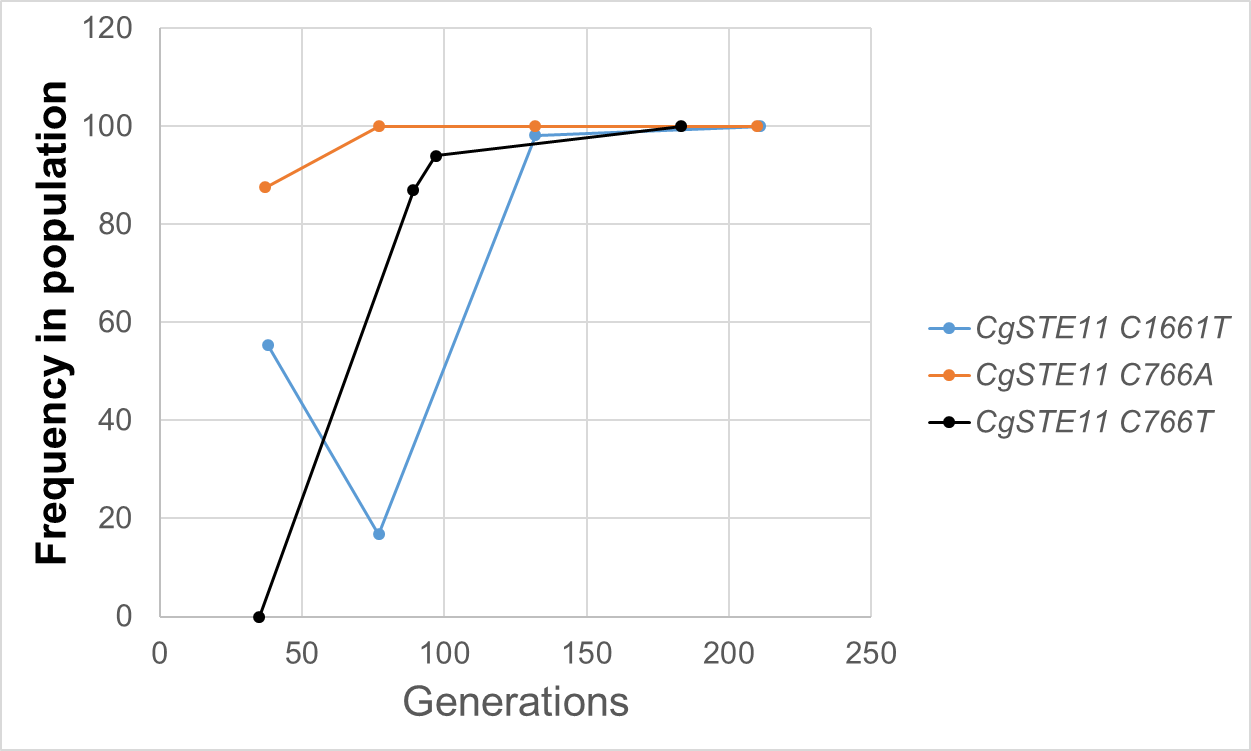
**

**Supplementary Figure S5.** Changes in population frequency of *CgSTE11* variants over time during the parallel adaptive evolution. Population frequencies of *CgSTE11* variants identified in population T1 (Blue), T2 (Red), and T3 (Black) were plotted over the time during the adaptation.

**Supplementary Figure S6**. The tolerance of *ste11∆* strain compared with ATCC 2001 and sMH081 towards (**a**) hyperthermal and (**b**) hyperosmotic stress. Hyperthermal tolerance assay was conducted using 1-hr shock at 50ºC. Hyperosmotic stress tolerance assay was conducted by plating cell dilutions on SC plates supplemented with 1 M NaCl. Images were taken 48 hours after incubation at 30ºC.
